# High-Performance Stable Field Emission with Ultralow Turn on Voltage from rGO Conformal Coated TiO_2_ Nanotubes 3D Arrays

**DOI:** 10.1038/srep11612

**Published:** 2015-07-08

**Authors:** Yogyata Agrawal, Garima Kedawat, Pawan Kumar, Jaya Dwivedi, V. N. Singh, R. K. Gupta, Bipin Kumar Gupta

**Affiliations:** 1 CSIR - National Physical Laboratory, Dr K S Krishnan Road, New Delhi, 110012, India; 2 Department of Physics, Kalindi College, University of Delhi, New Delhi, 110008, India; 3 Department of Chemistry, Pittsburg State University, Pittsburg, KS, 66762, USA

## Abstract

A facile method to produce conformal coated reduced graphene oxide (rGO) on vertically aligned titanium oxide (TiO_2_) nanotubes three dimensional (3D) arrays (NTAs) is demonstrated for enhanced field emission display applications. These engineered nano arrays exhibit efficient electron field emission properties such as high field emission current density (80 mA/cm^2^), low turn-on field (1.0 V/μm) and field enhancement factor (6000) with high emission current stability. Moreover, these enhancements observed in nano arrays attribute to the contribution of low work function with non-rectifying barriers, which allow an easy injection of electrons from the conduction band of TiO_2_ into the Fermi level of reduced graphene oxide under external electric field. The obtained results are extremely advantageous for its potential application in field emission devices.

Recently, high quality field emitters have gained importance owing to their potential for reliable integration into optoelectronic devices. Electron sources are essential elements in a variety of applications that include microwave amplifiers, parallel electron beam microscopes, x-ray sources and flat panel display technology^1^,^2^,^3^. One dimensional (1D) vertically-organized nanostructures (e.g. nanowires^4^,^5^,^6^, nanotubes^7^,^8^,^9^, nanobelts^10^,^11^ and nanoneedles^12^) are considered to be excellent field emission (FE) based electron emitters for delivering high current density at a low applied potential due to their high aspect ratios, high-field enhancement factor and low work functions. It is well established that the nanostructures having sharp tips can reduce the strength of turn-on electric fields by several orders of magnitude and decreases the barrier width due to the enhance local electric field at these tips. As a result, these nanostructure materials exhibited the excellent electron emission characteristics^13^. Among these 1D nanostructure materials, diamond based and carbon nanotubes (CNTs) have exhibited good FE performances owing to relatively low emission threshold field and high thermal and electrical conductivity^8^,^14^,^15^. However, further development of FE emitters depends critically on the challenging task of growing CNTs with specific properties as well as in current optoelectronic devices. In addition, higher work function, lack of adequate long-term or high-temperature FE stabilities and unsatisfactory mechanical properties have hindered the development of these materials for practical applications.

Wide band-gap semiconductors including TiO_2_^16^, MoO_3_^5^, SiC^17^, ZnO^12^, WO_3_^18^ and similar materials have also attracted much interest for their favorable FE properties because of their low electron affinity as well as better chemical stability. The band-bending effect of wide band gap semiconductors allows the field emission by lowering the surface barrier and bringing more electrons to the bottom of the conduction band. Among these materials, titanium oxide (TiO_2_), as a wide-band-gap (~ 3.1 – 3.2 eV) semiconductor, has been extensively studied because of its long-term thermodynamic stability, low cost, non-toxicity, strong oxidizing power and its optical as well as electrical properties. Hence, the vertically aligned TiO_2_ nanotube 3D arrays (NTAs) have created significant interest for good FE properties because of their sharp tips, low work function (4.4 eV), high aspect ratios, vertical orientation, tunable mesopore size, large internal surface area, convenient recycling and direct path for electron transport are considered having important FE properties^9^,^19^,^20^,^21^. However, the studies carried out of FE properties of TiO_2_ nanostructures have still been rather inadequate due to the limited success in reliable synthesis of conductive arrays. For better performance and economical cost of field emitter materials, it is important to look for carbon based nanostructure materials having high surface area. In this exploration, specially, the 2D (two dimensional) nanomaterials (e.g. graphene) can be a better alternative, which can be easily integrated with TiO_2_ at nanoscale to form hybrid materials for enhanced FE property.

Graphene with unique 2D π-π conjugated structure and a super strong form of carbon, has been regarded as a component of devices in recent years owing to its high electrical and thermal conductivity (~5,000 Wm^−1^K^−1^), good mobility of charge carriers (~200,000 cm^2^ V^−1^ s^−1^), superior chemical stability, high specific surface area (~2,630 m^2^ g^−1^) and sharp edges^22^,^23^,^24^ as well as its potential applications in field emission^25^, solar cells^26^, gas sensors^27^, transparent conducting electrodes^28^ and photocatalysts^29^. Graphene oxide (GO) is an excellent system with oxygen containing functional groups attached to the basal plane and edges, which makes it insulating and hydrophilic. These functional groups reduce the interaction energy between the graphene layers and thus make it dispersible in aqueous media. Graphene exhibits higher emission currents with lower external electric field and provides the large injection carriers^24^,^30^,^31^. Therefore, it can be a better electron-transport material than other carbon based materials^32^.

The graphene hybrid nanostructures have been extensively explored since the past decade for its highly-efficient field emission performances^33^,^34^,^35^,^36^,^37^. But in particular, the conformal coating of reduced graphene oxide (rGO) on vertically aligned TiO_2_ NTAs hybrid structure for FE studies has not been demonstrated till date. The combination of vertically aligned TiO_2_ NTAs with rGO is expected to expedite the development of various flexible devices. This also extends the application scopes and reinforces the properties of graphene and TiO_2_ materials. The conformal coating of rGO on TiO_2_ NTAs creates an additional 2D interface on TiO_2_ 3D NTAs thereby enhances the electron transport at low turn-on voltage. Thus, there is a growing interest in coupling the rGO-TiO_2_ NTAs to obtain an improved FE performance of TiO_2_ for the development of highly efficient cost-effective FE devices. This improvement is attributed to major factors as enlarged absorption region, narrow band gap of TiO_2_, enhanced electronic transfer and high surface area.

Herein, we report an approach to develop rGO-TiO_2_ NTAs hybrid nanostructures as efficient field emitters. In the investigations, vertically aligned TiO_2_ NTAs were grown on titanium (Ti) substrates via anodic oxidation method and then conformal coating of rGO was transfer onto TiO_2_ NTAs. It shows much improved FE properties than those obtained from pure TiO_2_ NTAs and rGO nanostructures. The morphology, dimensions and structural parameters of TiO_2_ NTAs are easily controlled by anodic oxidation parameters such as anodic voltage, oxidation time and electrolyte composition^38^,^39^,^40^. An anodic oxidation process has been used extensively for the rapid production of aligned TiO_2_ nanotubes because it has a good controlled pore size, uniformity and conformability over large areas. This is a facile process at economic cost and the desired properties can easily be obtained by tuning the dimensions. Moreover, in the present method, TiO_2_ nanotube has been formed on Ti sheet with a chemical bond between the oxide and Ti sheet. TiO_2_ nanotubes are strongly attached with Ti substrate, which provides convenience for TiO_2_ reusability. The field emission properties of TiO_2_ NTAs were investigated before and after being modified with rGO conformal coating and it was found that rGO conformal coated TiO_2_ NTAs have low turn-on field, high current density and uniform emission with better stability over a large area as compared to as-synthesized TiO_2_ NTAs. It is being demonstrated here that the incorporation of rGO through conformal coating on TiO_2_ NTAs greatly facilitates large surface area and charge carrier dynamics and improves the FE performance compared to other nanostructures such as commercial TiO_2_ nanoparticles (NPs), as-synthesized TiO_2_ NTAs and annealed TiO_2_ NTAs samples, which is not reported so far. The schematic presentation of synthesis process for highly oriented architecture of TiO_2_ NTAs via anodization technique with conformal coating of rGO on highly oriented annealed TiO_2_ 3D NTAs samples (40 V, 4 hours, 500 °C) is shown in Fig. 1.

## Results

The crystallinity and phase of samples were analyzed by X-ray diffraction technique. Fig. 2(a–c), shows the XRD pattern of as-synthesized TiO_2_ NTAs (Fig. 2a), annealed TiO_2_ NTAs (Fig. 2b) and conformal coated rGO on annealed TiO_2_ NTAs hybrid structures (Fig. 2c). The quantitative analysis of Fig. 2 (a,b) shows that all diffraction peaks correspond to TiO_2_ and Ti substrate and represents the tetragonal crystal structure with space group S.G. 14_1_/amd (141). The typical diffraction peak (101) centred at 25.1° indicates the TiO_2_ anatase phase (JCPDS No. 21–1272), which is formed after annealing at 500 °C for 2 hours. The peaks observed at (101), (103), (004), (112), (200), (105), (211), (213), (116) and (220) correspond to TiO_2_ anatase phase. It can be noticed that overall crystal structure becomes greatly refined after annealing. The lattice parameters were calculated from the observed d-values through a least square fitting method using computer program ‘unit cell refinement software’. These lattice parameter values are a = b = (3.7852 ± 0.0029) Å, c = (9.5139 ± 0.0067) Å for as-synthesized TiO_2_ NTAs and a = b = (3.79632 ± 0.0091) Å, c = (9.5832 ± 0.0097) Å for annealed TiO_2_ NTAs samples. The average domain size for as-synthesized and annealed TiO_2_ NTAs samplesare 105 nm and 121 nm with respect to (101) plane, which are estimated by using Scherer’s formula. The TiO_2_ NTA domains increase in size from 105 to 121 nm after the annealing process. The XRD pattern of as-synthesized rGO nanosheets is shown in Figure S1 (see Supplementary Information), where (002) and (101) planes confirm the graphitic nature with hexagonal phase (JCPDS No. 75–1621). There is no obvious difference in the TiO_2_ NTAs phase in annealed TiO_2_ NTAs (Fig. 2b) and conformal coated rGO on annealed TiO_2_ NTAs hybrid structure (Fig. 2c), indicating that the crystalline structure of TiO_2_ NTAs was not influence by the deposition of rGO nanosheets on TiO_2_ NTAs. However, no diffraction peak corresponding to rGO is observed in the hybrid structure, which may be because of the ultrathin nature of the rGO nanosheets layer coated on TiO_2_ NTAs. Raman spectroscopic measurement was used to characterize the reduction of graphene oxide (GO) as this process is very sensitive to crystallinity and microstructure of the materials. It was employed to confirm the formation of TiO_2_ anatase phase and the existence of rGO nanosheets on the surface of conformal coated rGO on annealed TiO_2_ NTAs hybrid structure. Figure 2d shows the Raman spectra of annealed TiO_2_ NTAs (Fig. 2d (i)) and conformal coated rGO on annealed TiO_2_ NTAs hybrid structure (Fig. 2d (ii)). Fig. 2d (i) shows the Raman peaks at 395, 516 and 640 cm^−1^ corresponding to the B_1g_, B_1g_ and A_1g_ and E_g_ modes of anatase TiO_2_^41^ respectively, which are consistent with the results of XRD. The Raman spectrum of as-synthesized TiO_2_ NTAs is shown in Figure S2 (a) (see Supplementary Information) in which all the three peaks (at 395, 516 and 640 cm^−1^) are observed. It confirms the formation of anatase phase. It can be easily observed that the peaks intensity is enhanced after annealing treatment (Fig. 2d (i)). The Raman spectrum of conformal coated rGO on annealed TiO_2_ NTAs hybrid structure is shown in Fig. 2d (ii). In addition to different TiO_2_ modes, the other new vibration mode D-band at 1354 cm^−1^and G band at 1600 cm^−1^ are also observed. These peaks correspond to the D and G band of rGO, which are attributed to the breathing mode of the k point photons of A_1g_ symmetry and first order scattering of E_2g_ phonon of the sp^2^ C atoms, respectively^42^. This confirmed the presence of graphene oxide in this conformal coated rGO on TiO_2_ NTAs hybrid structure. All other peaks (Fig. 2d (ii)) are due to the TiO_2_ NTAs. The intensity ratio of D to G band (I_D_/I_G_) has been proposed to be an indication of disorder in the graphene or rGO nanosheets and a low ratio indicates a greater disorder arising from structural defects. The conformal coated rGO on TiO_2_ NTAs has an intensity ratio (I_D_/I_G_) near to 0.940, suggesting that increased defects are brought by the reduction of graphene oxide^43^. The raman spectra of as-synthesized rGO nanosheets is shown in Figure S2 (b) (see Supplementary Information). Such characteristics demonstrate that the rGO nanosheets have direct evidence of conformal coating of rGO on the TiO_2_ NTAs.

The scanning electron microscope (SEM) and transmission electron microscope (TEM) were utilized to characterize the morphologies of as-synthesized, annealed TiO_2_ NTAs and conformal coated rGO on TiO_2_ NTAs hybrid structure. The typical SEM micrographs of as-synthesized and annealed TiO_2_ NTAs samples at anodization voltage 40 V for 4 hours are shown in Figure S3 (see Supplementary information). Figures S3 (a-b) exhibits the lateral and top view of as-synthesized TiO_2_ NTAs. Figures S3 (c-d) represents the lateral and top view of annealed TiO_2_ NTAs. It can be seen that both the samples consist of uniform open nanotubes of TiO_2_ with an average outer diameter of ~110 nm and maximum length of ~2 μm. The alignment is not disturbed after annealing; rather it helps to improve the crystallinity of sample, which is confirmed by XRD results. The typical SEM micrographs of as-synthesized TiO_2_ NTAs at different anodization voltage of 30, 40 and 50 V for 4 h anodization time are shown in Figure S4 (see Supplementary Information). The lateral view of as-synthesized TiO_2_ nanotube arrays sample for different anodization time intervals 1.5 and 2.5 h and the top view of TiO_2_ nanotube arrays sample at different anodization voltage of 30 and 50 V are shown in Figure S5 (see Supplementary Information). The bottom views of highly dense TiO_2_ NTAs after annealing at 500 °C for 2 hours is shown in Figure S6 (see Supplementary Information).

Figure 3(a) represents the TEM image of as-synthesized TiO_2_ NTAs and Fig. 3(b) exhibits the single TiO_2_ nanotube with outer diameter of ~110 nm, which is in good agreement with the SEM results. Figure 3(b) is the magnified version of Fig. 3(a), where microstructure of a single TiO_2_ nanotube can be easily seen in details. Fig. 3(c,d) shows the TEM/HRTEM images of vertically aligned TiO_2_ NTAs annealed at 500 °C for 2 hours. Figure 3(c) shows the TEM image of an isolated TiO_2_ nanotube with ~112 nm in diameter. It can be observed that after annealing, there is a slight variation in the outer diameter of TiO_2_ nanotube. The SAED pattern (inset of Fig. 3c) confirms the crystalline nature of TiO_2_ NTAs. The SAED ring pattern corresponding to (101), (200) and (004) lattice planes reveals the presence of TiO_2_ anatase phase. All the indexed planes are also observed in the XRD pattern of annealed TiO_2_ NTAs. Figure 3(d) demonstrates the HRTEM image of TiO_2_ nanotube having high quality lattice fringes without any distortion, which clearly demonstrates clear lattice fringes of TiO_2_ nanotubes after annealing process. The estimated interplanar spacing of adjacent lattice fringes is about ~0.35 nm, which corresponds to the (101) plane of anatase TiO_2_. The TEM and HRTEM images of TiO_2_ NTAs annealed at 500 °C for 2 hours, is shown in Figure S7 (see Supplementary Information). The TEM and SEM images of as-synthesized rGO nanosheets are shown in Figure S8 (see Supplementary Information), which reveals that the rGO nanosheets are composed of few layers of graphene.

In order to explore the surface morphology of rGO coated TiO_2_ NTAs, SEM study is carried out and results are shown in Fig. 3(e). A thin shadow of graphene around the TiO_2_ nanotube can be seen as marked by arrow. Additionally, the XPS studies on conformal coated rGO on annealed TiO_2_ NTAs were also conducted to find out the purity and chemical composition of TiO_2_ nanotubes. The XPS spectrum of conformal coated rGO on annealed TiO_2_ NTAs hybrid structure is shown in Figure S9 (see Supplementary Information) and inset clearly shows the core level spectrum of Ti. In the XPS spectrum, signals corresponding to titanium, oxygen and carbon are observed. No other signals are detected, which shows the high purity of as-synthesized conformal coated rGO on annealed TiO_2_ NTAs. The typical TEM image of conformal coated rGO on annealed TiO_2_ has been shown in Fig. 3(f). The TEM micrograph reveals clear microstructural information about the conformal coating of rGO on TiO_2_ nanotubes in the hybrid structure. The estimated number of rGO layers is simply calculated by the difference between the diameter of TiO_2_ nanotubes before and after coating of rGO on the TiO_2_ nanotubes arrays by using TEM image (from Fig. 3(c,f)). The obtained thickness of few layers of rGO conformally coated on TiO_2_ nanotubes is around 6 nm, which indicates that 16–17 layers of rGO are coated on TiO_2_ nanotubes. Further, the HRTEM image was taken to study the interface between the rGO and TiO_2_ and results are shown in Fig. 3(g). The HRTEM image has been taken from yellow marked region of Fig. 3(f). In Fig. 3(g), the yellow dashed line shows the interface between rGO and TiO_2_ lattices, from where we have estimated the lattice spacing. The graphene is well established for its binding capabilities with metal oxide particles as well as metals, such as TiO_2_ and Eu through covalent bonding or complexation without any aggregation^44^,^45^. In the present investigations, rGO nanosheets conformal coated on TiO_2_ NTAs appear to have strong interactions between them, which should lead to development of advanced hybrid materials to be used for various potential applications such as in field emission devices. Furthermore, the TiO_2_ nanotubes as well as elemental composition were evaluated by EDAX analysis. The spot EDAX measurement was performed with reduced beam spot size to enhance the signal to noise ratio. The EDAX spectrum was recorded on rGO conformal coated TiO_2_ NTAs area as shown in Fig. 3(f). The EDAX study reveals the presence of titanium, oxygen, copper and carbon element for rGO conformal coated TiO_2_ NTAs sample, as shown in Fig. 3(h). The small content of copper is from copper grid, which is used in TEM analysis. The atomic % ratio of titanium to oxygen is almost 1:2 as expected in the TiO_2_ molecule.

The electron field emission involves extraction of electrons from the NTAs by quantum tunneling through the surface potential barrier^46^. The field emission characteristics (field emission current density (*J*) as a function of applied electric field (*E*)) at a sample to cathode distance of 100 μm for conformal coated rGO on annealed TiO_2_ NTAs hybrid structure, annealed TiO_2_ NTAs, as-synthesized TiO_2_ NTAs, rGO-commercial TiO_2_ NPs, commercial TiO_2_ NPs, rGO-Ti sheet, rGO and Ti sheet samples are shown in Fig. 4(a). It is found that the emission current density exponentially increases with increase in the applied field for all the samples. An emission current density of 80 mA/cm^2^ at 230 V is obtained for conformal coated rGO on annealed TiO_2_ NTAs sample, which is the highest value compared to the other rGO-commercial TiO_2_ NPs, commercial TiO_2_ nanoparticles (NPs), as-synthesized TiO_2_ NTAs, annealed TiO_2_ NTAs samples, pure Ti sheet, rGO nanosheets and rGO on pure Ti sheet substrate samples. The obtained results suggest that rGO conformal coated on TiO_2_ NTAs hybrid structure is ultimate choice for better field emission characteristics. It may be due to the presence of large no. of delocalized π electrons on the surface of rGO which act as electron injection carriers^33^,^34^. Field emission current also depends on the aspect ratio of the TiO_2_ nanotubes, which is very high in the present case. The field emission characteristics of conformal coated rGO on annealed TiO_2_ NTAs, annealed TiO_2_ NTAs, as-synthesized TiO_2_ NTAs and commercial TiO_2_ NPs samples are shown in Figure S10a (see Supplementary Information). It can be noticed that the turn-on field (E_to_) for conformal coated rGO on annealed TiO_2_ NTAs, annealed TiO_2_ NTAs, as-synthesized TiO_2_ NTAs and commercial TiO_2_ NPs are 1.0, 1.4, 3.7 and 4.8 V/μm, respectively (from Fig. 4a and S10a). The turn-on field E_to_ values follows the sequences E_to_ (conformal coated rGO on annealed TiO_2_ NTAs) < E_to_ (annealed TiO_2_ NTAs) < E_to_ (as-synthesized TiO_2_ NTAs) < E_to_ (commercial TiO_2_ NPs). Furthermore, the field emission characteristics of different as-synthesized samples of conformal coated rGO on annealed TiO_2_ NTAs (sample 1, sample 2, sample 3 and sample 4) are also examined to explore reproducibility and the results are shown in Figure S10b (see Supplementary Information). It can be noticed that all the samples show similar and consistent behaviour. In addition to the above, the field emission behaviour of rGO, rGO-Ti sheet, rGO-commercial TiO_2_ nanoparticles and conformal coated rGO on annealed TiO_2_ NTAs samples from 1^st^ to 4^th^ cycle run are shown in the Figure S11a-d (see Supplementary Information). All tested samples show better emission uniformity and a good reproducibility of field emission behaviour during the initial 4 cycle run. The conformal coated rGO on annealed TiO_2_ NTAs clearly demonstrate the higher current density at low turn-on field (80 mA/cm^2^, 1.0 V/μm) in compared to all other samples (annealed TiO_2_ NTAs, as-synthesized TiO_2_ NTAs, rGO-commercial TiO_2_ NPs, commercial TiO_2_ NPs, rGO-Ti sheet, rGO and Ti sheet; Fig. 4a). The stability of emission current is also evaluated at 230 V for conformal coated rGO on annealed TiO_2_ NTAs, as shown in Fig. 4b and it is found to be very stable and no significant change is observed over a time period of 120 min at a current density 80 mA/cm^2^. Thus, the conformal coated rGO on annealed TiO_2_ NTAs shows a good electrical contact between the TiO_2_ nanotubes and rGO as well as it provides a long term stability of field emission currents.

Field emission is generally analyzed using the Fowler–Nordheim (F-N) theory, which describes the tunneling of electrons through a potential barrier formed at the interface between a metal surface and vacuum^46^. According to F-N theory, the field emission current (*I*) or current density (*J*) is related to work function (*Ф*) of the material and external electric field (*E*) through the relation,




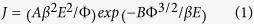




or




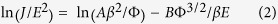




Where *J* is the current density, *E* is the applied field, *Ф* is the work function of the emitting materials (~4.4 eV for TiO_2_), *β* is field enhancement factor and A and B are constants with values of 1.56 × 10^−6^ (A V^−2^ eV) and 6.83 × 10^3^ (V μm^−1^ eV^−3/2^) respectively. The value of *β* is related to the emitter geometry, crystal structure, vacuum gap and spatial distribution of the emitter centres. The F-N plots of log_2_(*J*/*E*^2^) versus 1*/E* for different samples are shown in the Fig. 4c and different slopes for the TiO_2_ NTAs before and after being modified with rGO conformal coating are observed. Good linearity within the measurement range suggests that electron emission by samples follows the F-N plots and the emission is indeed due to a vacuum tunnelling process. Moreover, the work function (*Ф*) of as-synthesized samples was calculated using photoelectron emission (PEE) technique. The PEE spectra for conformal coated rGO on annealed TiO_2_ NTAs, annealed TiO_2_ NTAs, as-synthesized TiO_2_ NTAs and commercial TiO_2_ NPs samples are shown in Figure S12a (see Supplementary Information). The plausible schematic model of edge states and corresponding energy-band diagrams of field emission from conformal coated rGO on annealed TiO_2_ NTAs, annealed TiO_2_ NTAs, as-synthesized TiO_2_ NTAs and commercial TiO_2_ NPs samples is shown in Figure S12b (see Supplementary Information). It reveals that conformal coated rGO on annealed TiO_2_ NTAs consist of higher ratios of C–O–C ether chain edge states, which causes the potential barrier of electrons have to overcome in vacuum to be diminished, resulting in a lower work function of conformal coated rGO on annealed TiO_2_ NTAs hybrid structure. So electrons tunnel through near the top of the barrier and can easily pass across the full barrier width. The experimentally obtained work function value for conformal coated rGO on annealed TiO_2_ NTAs, annealed TiO_2_ NTAs, as-synthesized TiO_2_ NTAs and commercial TiO_2_ NPs are ~3.1 eV, ~3.3 eV, ~3.9 eV and ~4.4 eV, respectively. Thus, from the slope of F-N plots and calculated values of work function, we can easily estimate the field enhancement factors *β* from equation 2. It is ~6000, ~5000, ~700 and ~600 for conformal coated rGO on annealed TiO_2_ NTAs, annealed TiO_2_ NTAs, as-synthesized TiO_2_ NTAs and commercial TiO_2_ NPs, respectively. From the above results, we can observe that the field enhancement factor value is higher for conformal coated rGO on annealed TiO_2_ NTAs samples compared to the other previous reported TiO_2_ nanostructures^4^,^16^,^37^,^47^,^48^. This can be due to the combined effect of rGO and TiO_2_. The rGO provides an additional interface to the large curvature of TiO_2_ NTAs because 2D rGO nanosheets have a sufficient no. of the delocalized π electrons available^49^,^50^, which act as electron injection carriers and, as a result the difference between the Fermi levels of rGO and conduction band of TiO_2_ in rGO- TiO_2_ hybrid structure is reduced, consequently the work function also get reduced. The experimentally obtained work function of the rGO-TiO_2_ hybrid structure is less than that of the TiO_2_, which can be seen from Figure S12a. According to the results presented here, TiO_2_ NTAs possess moderate high performance field emission property, which is enhanced remarkably after being modified with conformal coating of rGO. It is mainly attributed to low work function and high aspect ratio. Thus, the introduction of rGO on the surface of TiO_2_ NTAs can increase the number of emitters and tunneling probability, which leads to higher field emission for the hybrid emitters. These results showed that the field emission properties of TiO_2_ NTAs can be tailored by conformal coating of rGO on its surface. The improved field emission characteristics in conformal coated rGO-TiO_2_ NTAs hybrid structures are attributed to the contribution of low work function of the metal and the field free vacuum (E_v_)) (Figure S12b). The ohmic contact with non-rectifying barriers allows electrons to be easily injected from the conduction band of TiO_2_ to Fermi level of rGO under external electric field. Then, the electrons go from TiO_2_ to rGO, then to vacuum through subsequent F-N tunneling under the low turn-on field. These results indicate the great shift of Fermi level towards higher energy, as shown in schematic diagram (Figure S12b). Therefore, compared to TiO_2_, a Fermi level with higher energy is observed for conformal coated rGO-TiO_2_ NTAs hybrid structures. This shifting improves both the conductivity and field emission properties of conformal coated rGO-TiO_2_ NTAs sample. This work demonstrates the approach to convert TiO_2_ nanotube arrays into conformal coated rGO-TiO_2_ NTAs hybrid structure. As a result, it creates more acceptor and donor states (both) above the valence band maximum and below the conduction band minimum in the band gap of TiO_2_ nanotube, which helps to reduce work function of hybrid structure (clearly shown in Figure S12a and S12b). Therefore, the conformal coated rGO-TiO_2_ NTAs is better hybrid structure for obtaining the high-performance field emission applications.

## Discussion

A highly-efficient method to produce hybrid structure of rGO nanosheets conformal coated on vertically aligned TiO_2_ nanotubes 3D arrays for enhanced field emission display applications has been successfully demonstrated. The structural characterization of rGO conformal coated annealed TiO_2_ NTAs exhibits the formation of a highly ordered 3D NTAs with a pure anatase phase and good crystallinity. SEM and TEM results indicated that the average diameter and length of TiO_2_ NTAs are about ~110 nm and ~2 μm respectively, at optimum anodization condition (anodization at 40 V for 4 h). The HRTEM image of TiO_2_ NTAs shows high quality lattice fringes without lattice distortion, which clearly demonstrates the improvement of crystal line quality of TiO_2_ NTAs after annealing.

The rGO conformal coated TiO_2_ NTAs exhibited high emission current and excellent field emission stability with a low turn on field compared to commercial TiO_2_ nanoparticles (NPs), as-synthesized TiO_2_ NTAs, annealed TiO_2_ NTAs samples, pure Ti sheet, rGO nanosheets and rGO on pure Ti sheet substrate samples. The linearity of the F-N plots confirms that the process is governed by the Fowler-Nordheim equation, based on tunneling electron emission. Thus, this simple, effective and robust approach provides new prospects to develop highly-efficient electron sources for stable and ultra low turn on field FE devices based on the rGO conformal coated TiO_2_ NTAs hybrid nanostructures.

## Methods

### Materials

The titanium (Ti) sheet (99.8% purity, size ~0.5 mm × ~20 mm × ~15 mm) was purchased from Sigma-Aldrich. Graphite flakes (SP-1 graphite, ~150 μm size) was purchased from Bay Carbon Corporation. Ammonium fluoride (NH_4_F), ethylene glycol (C_2_H_6_O_2_), hydrogen peroxide solution (H_2_O_2_), potassium permanganate (KMnO_4_) and all other reagents were of analytical grade (AR) and used as received without further purification. Double distilled water was used throughout the experiments.

### Synthesis of vertically aligned TiO_2_ 3D NTAs

**Figure 1:**
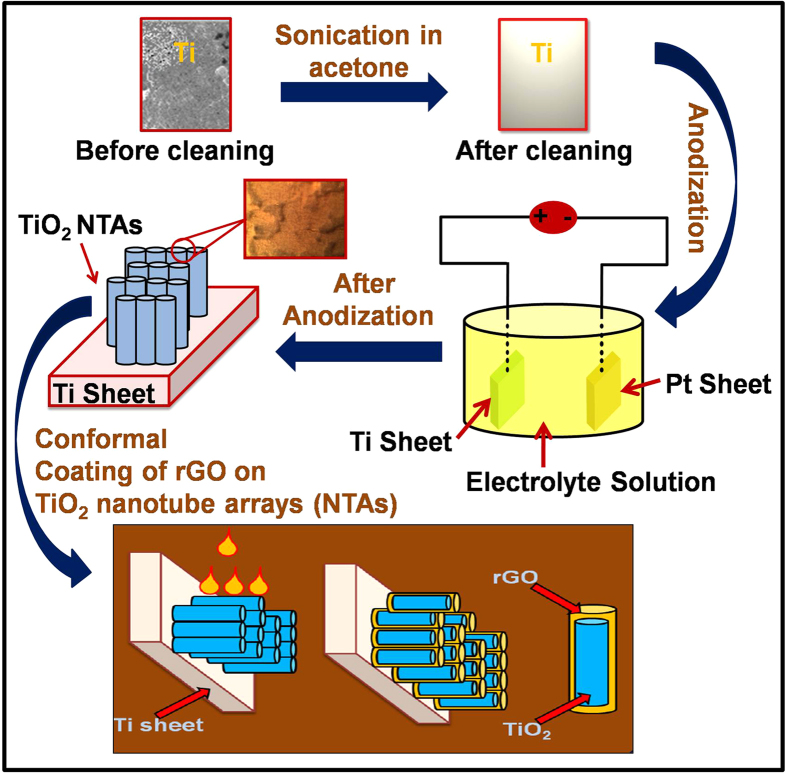
The synthesis process for highly oriented architecture of TiO_2_ NTAs via anodization technique with conformal coating of rGO on highly oriented annealed TiO_2_ 3D NTAs samples (40 V, 4 hours, 500 °C).

**Figure 2:**
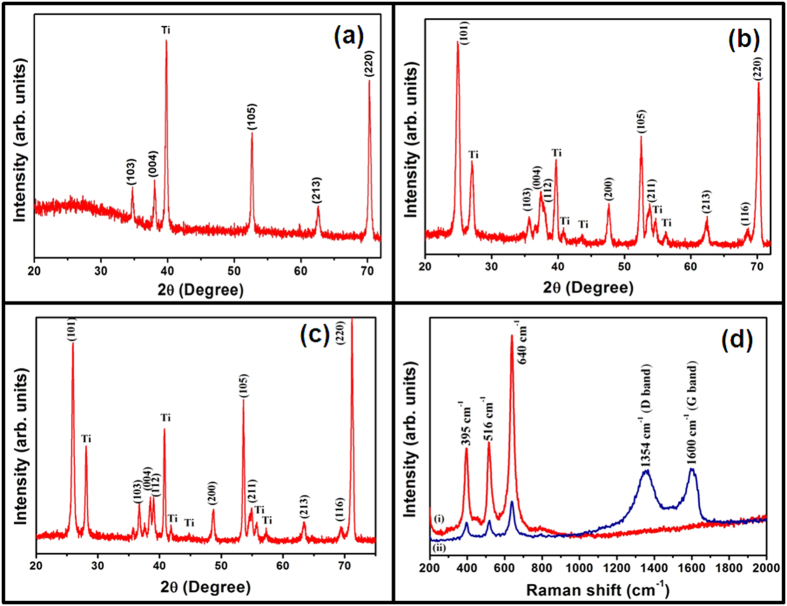
The XRD patterns of (**a**) as-synthesized TiO_2_ NTAs, (**b**) annealed TiO_2_ NTAs, (**c**) conformal coated rGO on TiO_2_ NTAs hybrid structure and (**d**) the Raman spectra of (i) annealed TiO_2_ NTAs and (ii) conformal coated rGO on annealed TiO_2_ NTAs hybrid structure.

**Figure 3:**
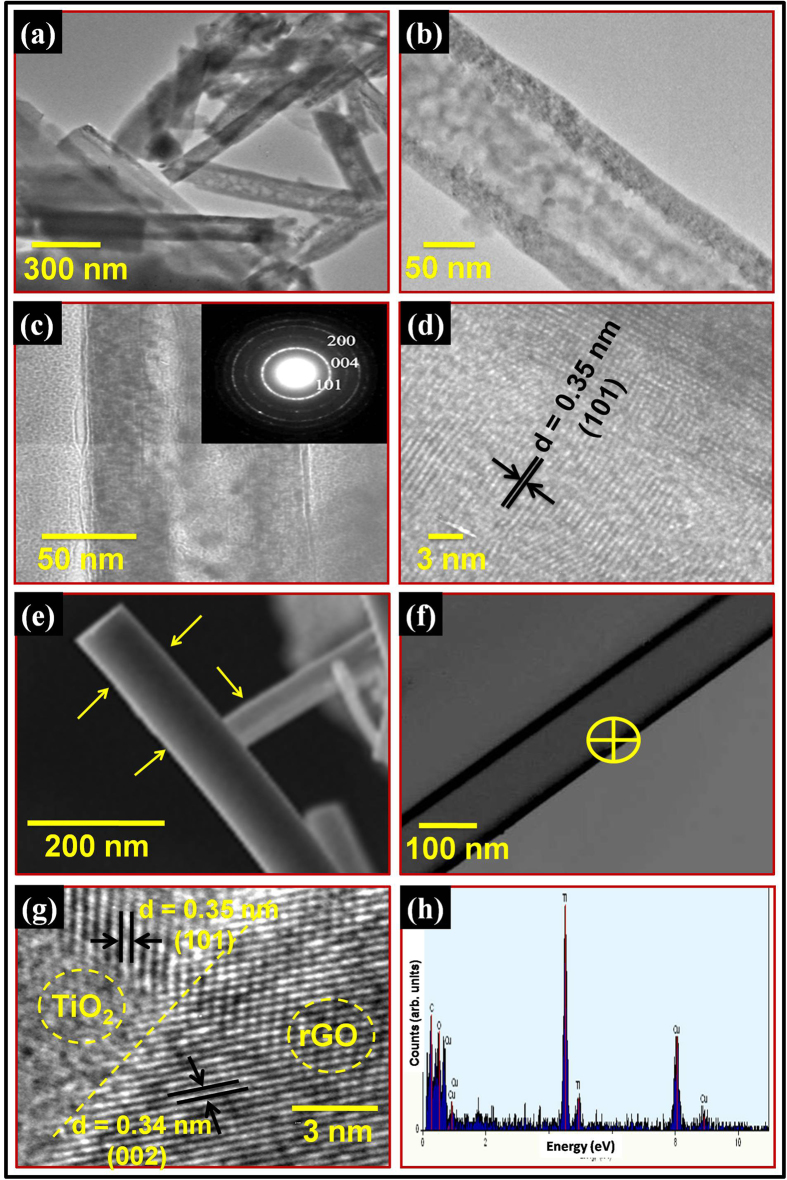
TEM images of (**a**) as-synthesized TiO_2_ NTAs, (**b**) magnified version of (**a**), (**c**) annealed TiO_2_ NTAs at 500 °C for 2 hour and inset shows the SAED pattern of annealed TiO_2_ NTAs and (**d**) HRTEM image of annealed TiO_2_ NTAs, (**e**) SEM, (**f**) TEM, (**g**) HRTEM images of conformal coated rGO on annealed TiO_2_ NTAs; where micrographs clearly evidence the conformal coating of rGO on annealed TiO_2_ NTAs and (**h**) EDAX pattern of conformal coated rGO on annealed TiO_2_ NTAs.

**Figure 4:**
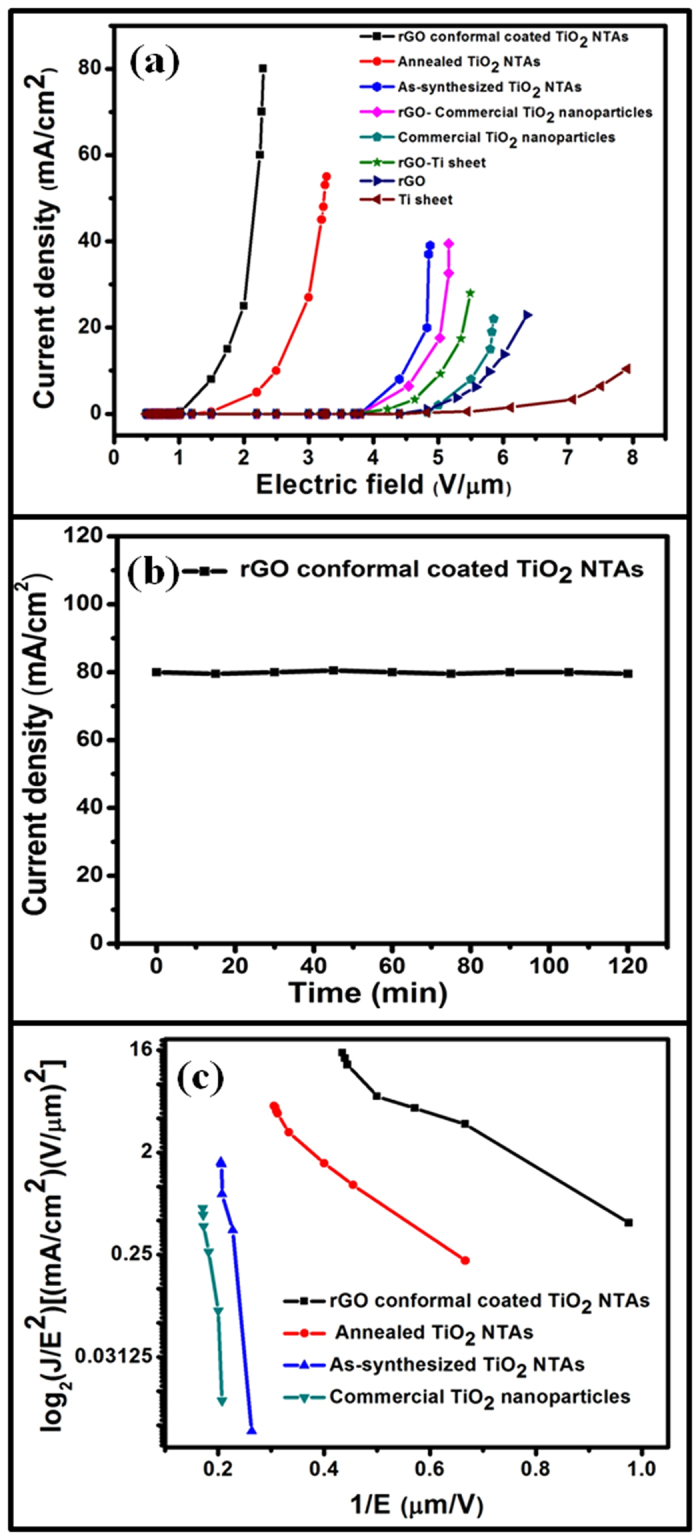
Field emission characteristics of typical field emission devices based on rGO-TiO_2_ NTAs hybrid nanostructures (**a**) Field emission characteristics of different field emission devices (conformal coated rGO on annealed TiO_2_ NTAs hybrid structure, annealed TiO_2_ NTAs, as-synthesized TiO_2_ NTAs, rGO-commercial TiO_2_ NPs, commercial TiO_2_ NPs, rGO-Ti sheet, rGO and Ti sheet samples), (**b**) stability of field emission currents from a typical field emission device (conformal coated rGO on annealed TiO_2_ NTAs hybrid structure) at 230 voltages and (**c**) Fowler-Nordheim characteristics curves for different field emission devices (conformal coated rGO on annealed TiO_2_ NTAs, annealed TiO_2_ NTAs, as-synthesized TiO_2_ NTAs and commercial TiO_2_ NPs).

Vertical aligned TiO_2_ 3D NTAs were fabricated by anodic oxidation of 0.5 mm thick Ti sheet. Prior to anodization, titanium sheets were first mechanically polished with different grades of emery papers and final finishing was done with zero grade paper. Then, Ti sheets were ultrasonically (frequency; 25 kHz) cleaned in acetone and ethyl alcohol for 10 minutes in each solution. This process was repeated three times to get nearly clean Ti-sheet and then dried in air at room temperature. The synthesis process for highly oriented architecture of 3D TiO_2_ nanotubes arrays using anodization technique is illustrated in Fig. 1. The electrochemical anodization of Ti sheet was carried out in a two-electrode cell, with a platinum foil as counter electrode, which was immersed in electrolytic solution in a beaker. The distance between the two electrodes was 2 cm. Both electrodes were placed parallel to keep constant flux lines or the uniform current between the electrodes. Two Cu-wires were used for making the connection through the electrodes. These electrodes were mounted on glass rods using rapid repair material (self polymerizing powder and liquid). Electrolyte was comprised of 0.3 wt% NH_4_F and 5.0 vol% deionized water in an ethylene glycol solution. The anodization procedure was carried out at different applied potential of ~30 V, ~40 V and ~50 V as well as for various time intervals of 1.5, 2.5, 3.5 and 4.0 hours to optimize the anodization process. The electrochemical experiments were carried out at room temperature under the assistance of magnetic stirring. After anodization, the samples were rinsed with deionized water to remove any unwanted ions on the surface of the TiO_2_ NTAs samples and dried in air. The optical images of as-synthesized TiO_2_ 3D NTAs samples at different anodization voltage as well as for various time intervals are shown in Figure S13 (see Supplementary Information). The detailed electrochemical conditions with calculated length and diameter of tubes are listed in Table TS1 and TS2 (see Supplementary Information). The surface morphology of as anodized samples was observed by scanning electron microscope and found that the optimum condition for anodization process with large outer diameter of tube is occurred at 40 V for 4 hours. After optimizing the condition, the obtained amorphous nanotube arrays samples were annealed at 500 °C for 2 hours with heating and cooling rates of 2 °C/min to obtain pure anatase phase. The high*-*resolution optical micrograph image of TiO_2_ NTAs at 4 V anodization voltages and 4 hours time intervals at different scale is shown in Figure S14 (see Supplementary Information) at different scale.

### Synthesis of reduced graphene oxide nanosheets

Reduced graphene oxide nanosheets were as-synthesized by the oxidation of graphite flakes using improved method proposed and established by James Tour *et al.*^51^ 3.0 g graphite flakes was added in solution of H_2_SO_4_/H_3_PO_4_ (360:40 mL) and 18.0 g KMnO_4_, producing a slight exotherm at 35–40 °C temperature. The mixture was continuously stirred at 50 °C for 12 h and allowed to cool to room temperature. It was poured onto ice (400 mL) and treated with hydrogen peroxide solution (H_2_O_2_, 30 wt%, 3 mL). The mixture was sifted through a metal U.S. Standard testing sieve (W.S. Tyler, 300 μm) and filtered through polyester fiber (Carpenter Co.). Then, it was centrifuged (4000 rpm, 4 h) and the supernatant was decanted away. The remaining solid material was washed in succession with 200 mL of 30% HCl, water and ethanol. For each wash, the mixture was sifted through the U.S. Standard testing sieve and then filtered through polyester fiber with the filtrate being centrifuged and the supernatant decanted away. The remaining material after this extended, the multiple-wash process was coagulated with 200 mL of ether. The resulted suspension was filtered over a PTFE membrane with 0.45 μm pore size. The powder obtained on the filter was dried overnight at room temperature under vacuum and 5.8 g graphene oxide product was obtained. Finally, 0.1 g graphene oxide was dispersed into 100 mL distilled water via ultrasonication and then NaBH_4_ was added to reduce the graphene oxide nanosheets to graphene nanosheets at 80 °C.

### Fabrication of reduced graphene oxide conformal coated TiO_2_ 3D nanotubes arrays

The as-synthesized rGO 2D nanosheets were ultrasonically dispersed into ethanol, followed by ultrasonication at 25 kHz frequency for 1 hour to form a homogeneous suspension with a concentration of 0.05 mg/mL. The resulted solution was drop-casted on the samples perpendicular to the orientation of the NTAs (40 V, 4 hours, 500 °C) and dried in air, as shown in Fig. 1. It formed Ti-O-C bonding between TiO_2_ and rGO, which is further confirmed by XPS. The dilution of rGO played a critical role to obtain enhanced FE properties as well as easy to coat conformably around the nanotube walls. Similarly, the rGO conformal coated TiO_2_ NTAs samples are prepared four times for reproducibility test and are labeled as sample 1, sample 2, sample 3 and sample 4.

### Characterization

For phase identification and gross structural analysis, the structural characterization was performed using X-ray diffractometer (XRD, Rigaku: MiniFlex, Cu Kα_1_, λ = 1.5406 Å). The surface morphology, length and diameter of TiO_2_ nanotubes were determined by scanning electron microscopy (SEM, Model No. EVO-MA 10 VPSEM). The microstructural studies were carried out using high-resolution transmission electron microscopy (HRTEM, Model No. Technai G20-twin, 300 kv with super twin lenses having point and line resolution of 0.144 nm and 0.232 nm, respectively) equipped with energy dispersive x-ray analysis (EDAX) facility. Raman spectra were obtained using Renishaw InVia Raman spectrometer, UK with an excitation source of 514.5 nm. The XPS analysis was carried out in an ultra-high vacuum (UHV) chamber equipped with a hemispherical electron energy analyzer (Perkin Elmer, PHI1257) using non-monochromatized Al Kα source (excitation energy of 1486.7 eV) with a base pressure of 4 × 10^−10^ torr at room temperature. The work function has been evaluated through open-counter photoelectron emission (PEE) spectroscopy system.

### Field emission measurements

The field emission measurements were carried out at room temperature under a vacuum of ~10^−6^ torr. A rod like copper probe with a cross section of about 0.6 mm^2^ was served as an anode and all samples; conformal coated rGO on annealed TiO_2_ NTAs, annealed TiO_2_ NTAs, as-synthesized TiO_2_ NTAs and commercial TiO_2_ NPs on the Ti substrate were fixed onto ITO as the cathode under same condition. Field emission measurements were performed in high vacuum to prevent the rGO from absorbing oxygen. The spacing between the electrodes was maintained at 100–500 μm, 100 μm was kept as an optimum distance. A dc voltage sweep from 300 to 1100 V was applied to the samples in steps of 20 V to generate the electric field (*E*). The emission current was monitored by an electrometer (Keithley 6514) with picoampere sensitivity.

## Additional Information

**How to cite this article**: Agrawal, Y. *et al.* High-Performance Stable Field Emission with Ultralow Turn on Voltage from rGO Conformal Coated TiO_2_ Nanotubes 3D Arrays. *Sci. Rep.* **5**, 11612; doi: 10.1038/srep11612 (2015).

## Supplementary Material

Supplementary Information
