# Supplementary material for: High-Performance Stable Field Emission with Ultralow Turn on Voltage from rGO Conformal Coated TiO2 Nanotubes 3D Arrays
Source: Sci Rep. 2015 Jul 8;5:11612. doi: 10.1038/srep11612 (PMC4495390; doi:10.1038/srep11612)
Supplement: Supplementary Information [file srep11612-s1.docx]

**Supplementary Information**

**High-Performance Stable Field Emission with Ultralow Turn on Voltage from rGO Conformal Coated TiO_2_ Nanotubes 3D Arrays**

Yogyata Agrawal^1^, Garima Kedawat^2^, Pawan Kumar^1^, Jaya Dwivedi^1^, V. N. Singh^1^, R. K. Gupta^3^ and Bipin Kumar Gupta*^,1^

^1^CSIR - National Physical Laboratory, Dr K S Krishnan Road, New Delhi, 110012, India, ^2^Department of Physics, Kalindi College, University of Delhi, New Delhi, 110008, India, ^3^Department of Chemistry, Pittsburg State University, Pittsburg, KS, 66762, USA

*E-mail:[bipinbhu@yahoo.com](mailto:bipinbhu@yahoo.com) (B.K.G.)


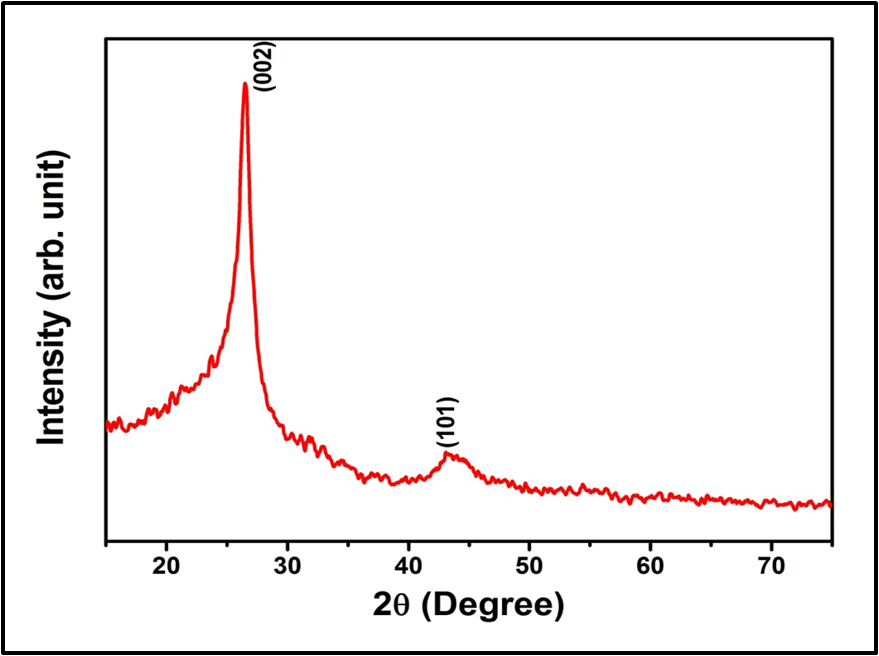


**Figure S1.** The XRD pattern of as-synthesized rGO nanosheets.


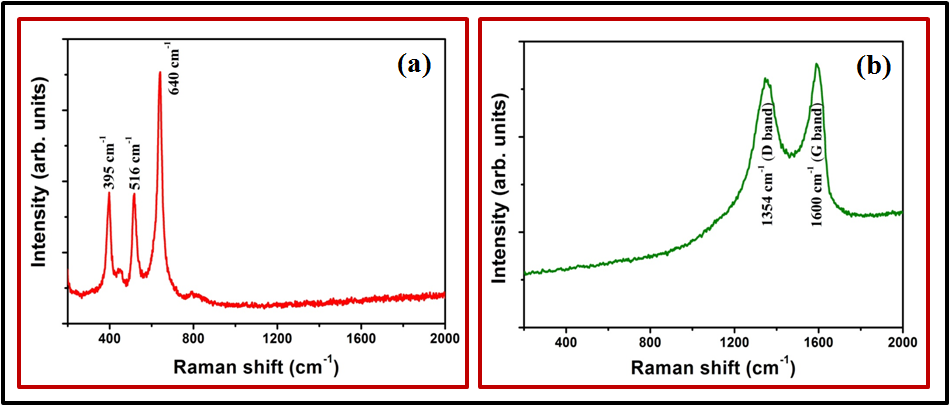


**Figure S2:** The Raman spectra of as-synthesized (a) TiO_2_ NTAs and (b) rGO nanosheets.


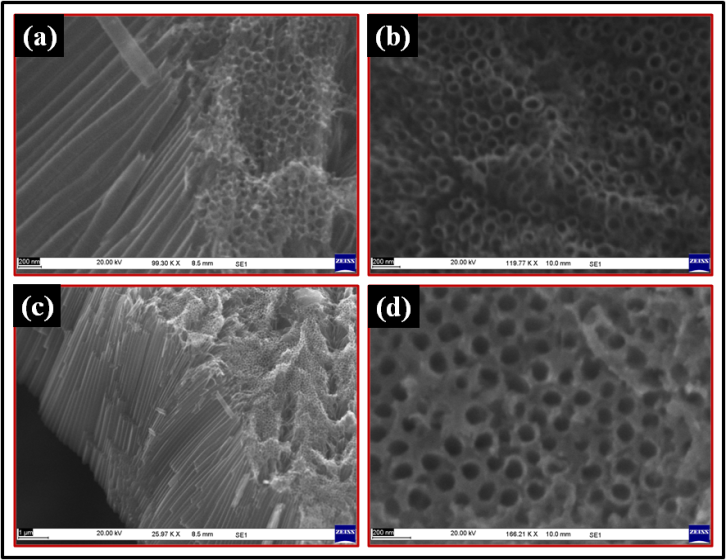


**Figure S3.** SEM images of the (a) lateral view of as-synthesized TiO_2_ 3D NTAs, (b) top view of as-synthesized TiO_2_ 3D NTAs, (c) lateral view of annealed TiO_2_ NTAs and (d) top view of annealed TiO_2_ NTAs at 500°C for 2 hour.


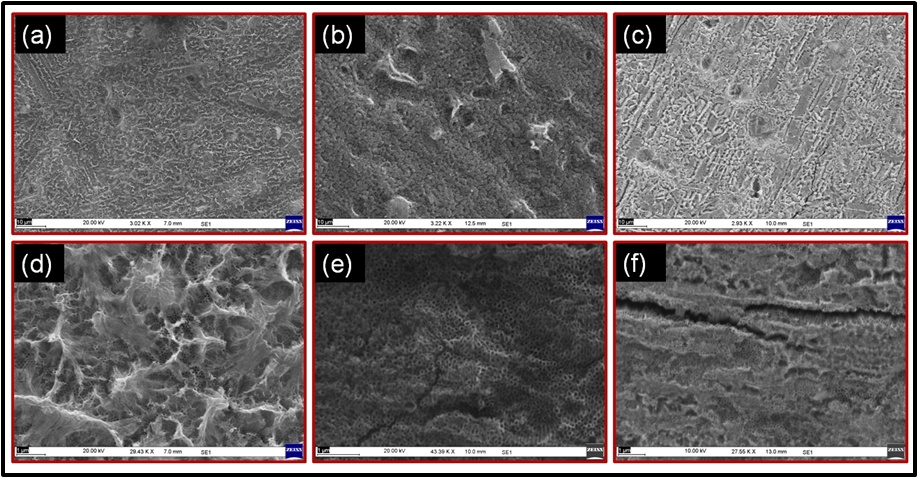


**Figure S4:** (a-c) the typical SEM micrographs of as-synthesized TiO_2_ nanotube arrays sample at different anodization voltage 30, 40 and 50 V, respectively, for 4h anodization time and (d-f) the magnified view of Figure S4(a-c).


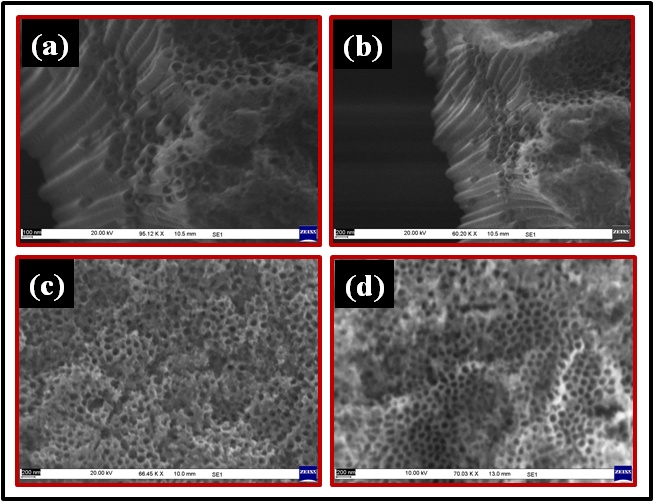


**Figure S5:** (a-b) exhibit the lateral view of as-synthesized TiO_2_ NTAs sample for different anodization time intervals 1.5 and 2.5 h and (c-d) represent the top view of TiO_2_ NTAs at different anodization voltage 30 and 50 V.


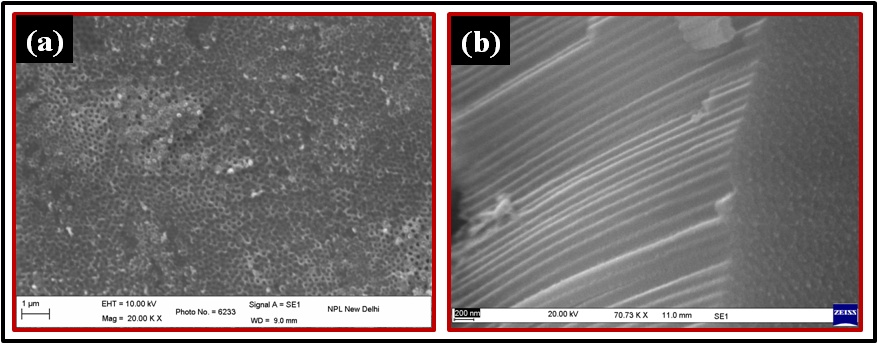


**Figure S6:** (a) bottom view of highly dense annealed TiO_2_ NTAs and (b) lateral bottom view of annealed TiO_2_ NTAs sample.


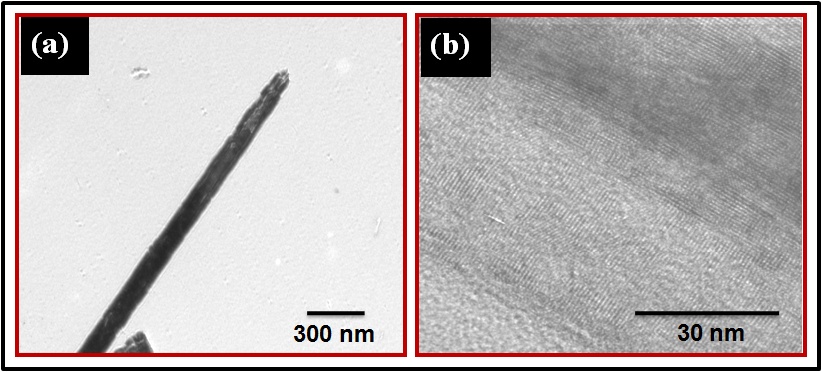


**Figure S7:** (a) TEM and (b) HRTEM images of annealed TiO_2_ NTAs at 500^°^C for 2 hours.


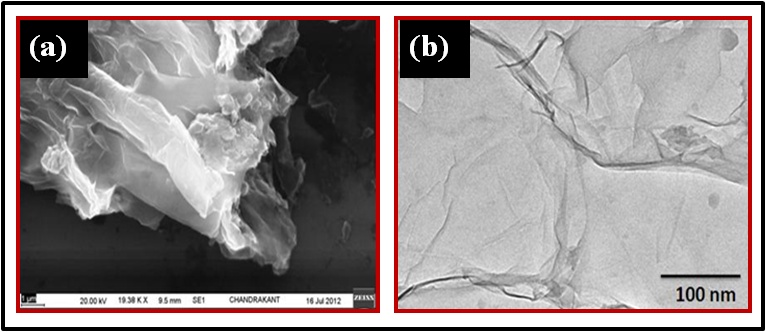


**Figure S8:** (a) SEM and (b) TEM images of as-synthesized rGO nanosheets.


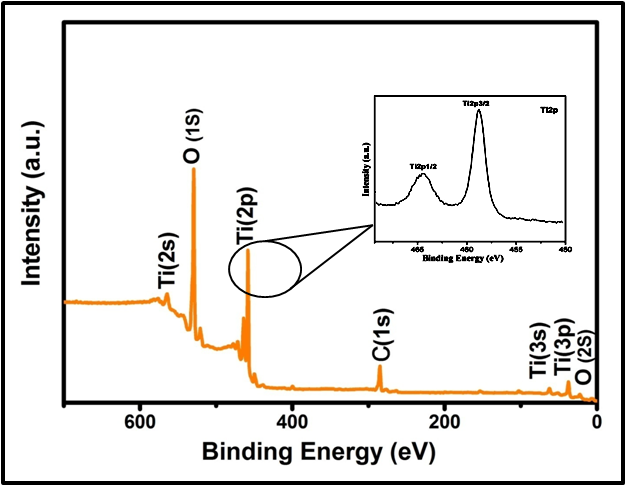


**Figure S9:** XPS spectrum of conformal coated rGO on annealed TiO_2_ NTAs hybrid structure and inset shows the core level spectrum of Ti.


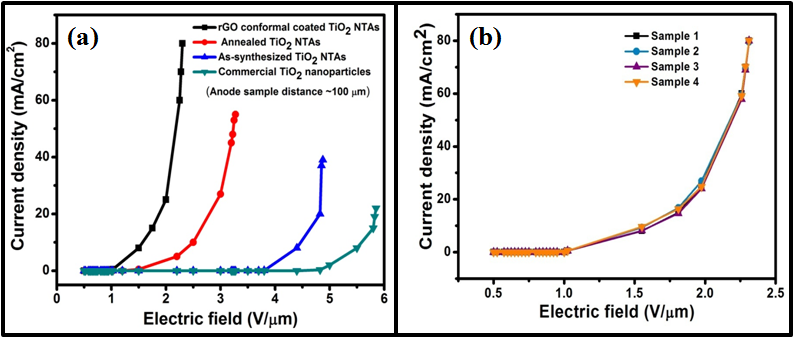


**Figure S10:** (a) Field emission characteristics of different field emission devices (conformal coated rGO on annealed TiO_2_ NTAs, annealed TiO_2_ NTAs, as-synthesized TiO_2_ NTAs and commercial TiO_2_ NPs) and (b) Field emission characteristics of different as-synthesized samples of conformal coated rGO on annealed TiO_2_ NTAs (sample 1, sample 2, sample 3 and sample 4). As evident from figure, all four samples are having almost similar FE behaviour indicating good reproducibility.


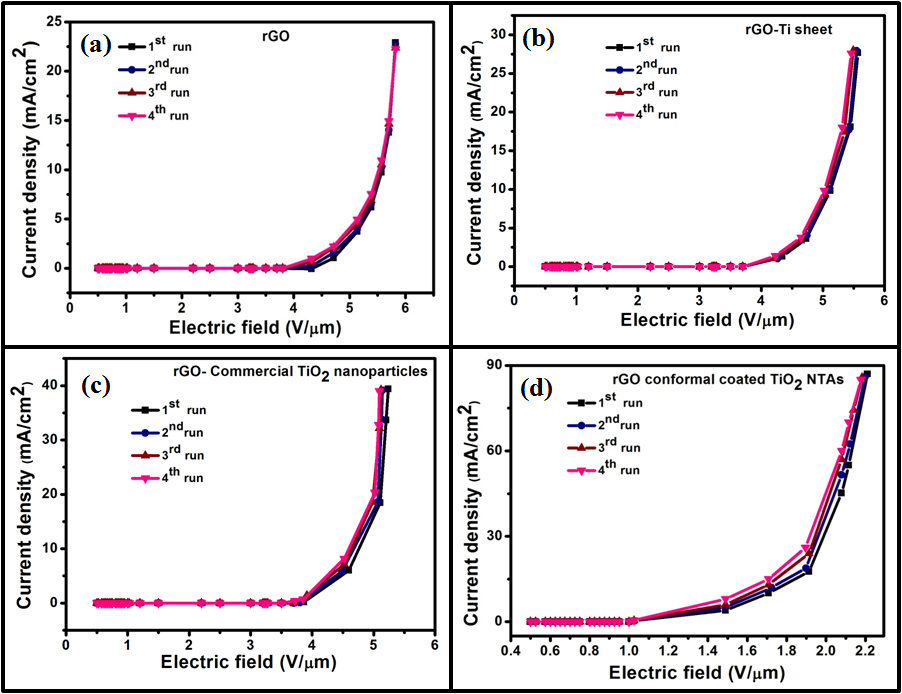


**Figure S11:** Field emission characteristics of (a) rGO, (b) rGO-Ti sheet, (c) rGO-commercial TiO_2_ nanoparticles and (d) rGO conformal coated TiO_2_ NTAs samples from 1^st^ to 4^th^ cycle run, showing better emission stability.

**
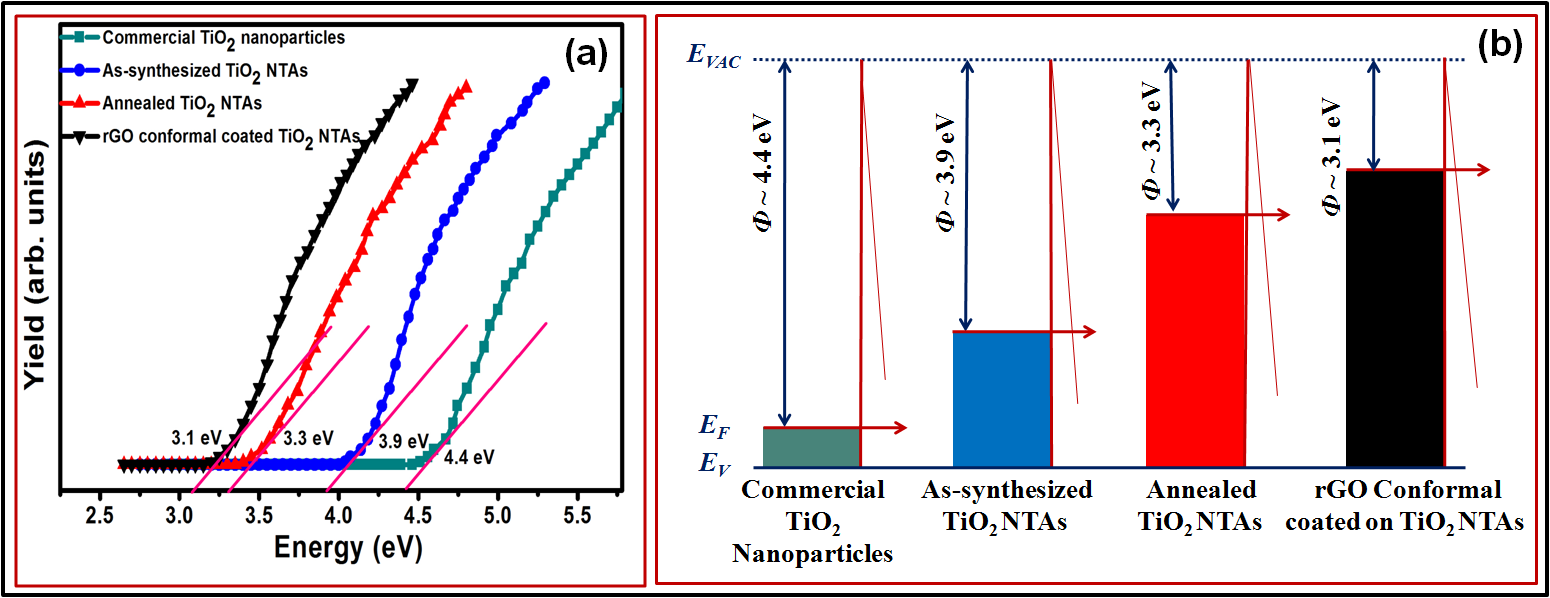
**

**Figure S12:** (a) PEE spectra for conformal coated rGO on annealed TiO_2_ NTAs, annealed TiO_2_ NTAs, as-synthesized TiO_2_ NTAs and commercial TiO_2_ NPs samples and (b) the plausible schematic model of edge states and corresponding energy-band diagrams of field emission from conformal coated rGO on annealed TiO_2_ NTAs, annealed TiO_2_ NTAs, as-synthesized TiO_2_ NTAs and commercial TiO_2_ NPs samples (E_VAC_: vacuum level, E_V_: the top of the valence band, E_F_: Fermi level, Ф: work function).

**.**

**
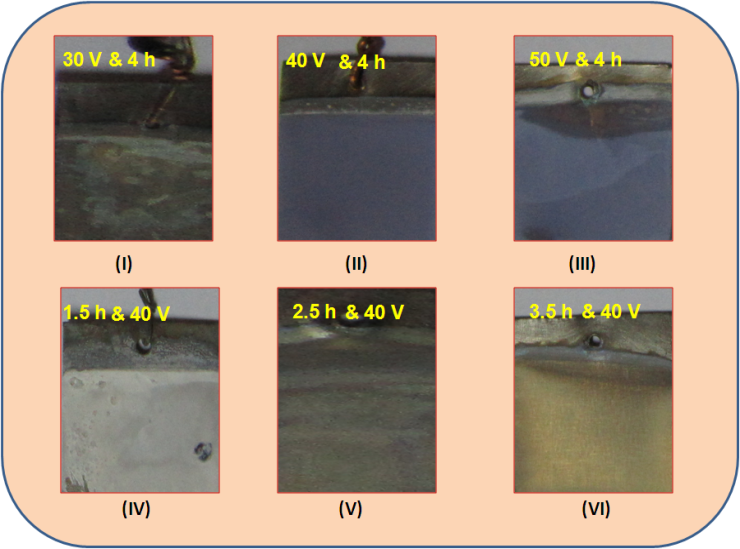
**

**Figure S13:** Optical photographs of as-synthesized TiO_2_ 3D NTAs samples at different anodization voltage as well as for various time intervals.

**
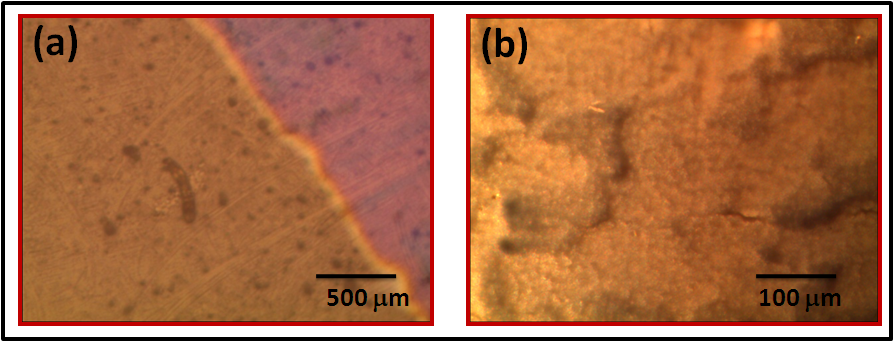
**

**Figure S14:** High-resolution optical micrograph images of TiO_2_ 3D NTAs at 4V anodization voltages and 4 hours time intervals having different scale (top surface).

**Table TS1: The electrochemical conditions with calculated length of TiO_2_ NTAs.**

**
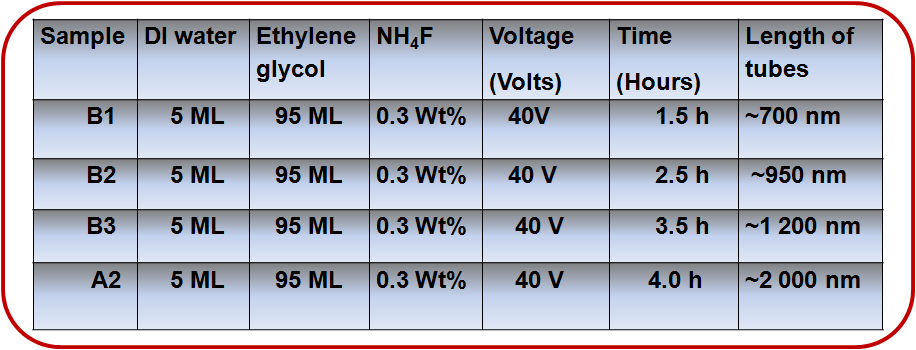
**

**Table TS2: The electrochemical conditions with calculated diameter of TiO_2_ NTAs.**

**
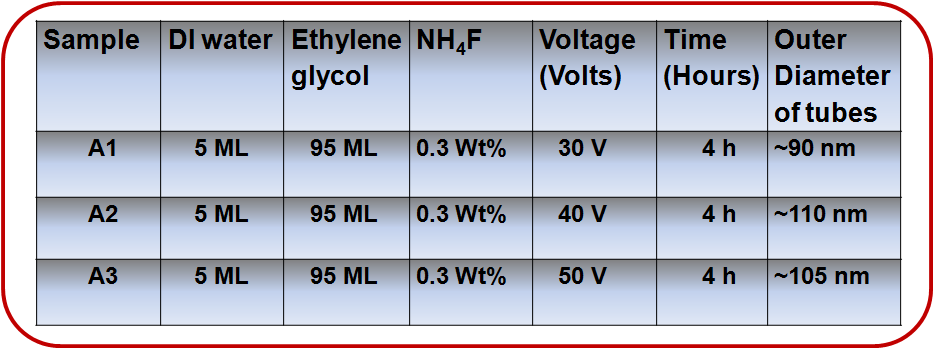
**
